# Supplementary material for: Inverse designed plasmonic metasurface with parts per billion optical hydrogen detection
Source: Nat Commun. 2022 Sep 30;13:5737. doi: 10.1038/s41467-022-33466-8 (PMC9525276; doi:10.1038/s41467-022-33466-8)
Supplement: Supplementary file 1 — Supplementary Information [file 41467_2022_33466_MOESM1_ESM.pdf]

Supplementary Information for

Inverse designed plasmonic metasurface with parts per billion optical hydrogen detection

Ferry Anggoro Ardy Nugroho[1,2,*], Ping Bai[3], Iwan Darmadi[4], Gabriel W. Castellanos[3], Joachim Fritzsche[4], Christoph Langhammer[4,*], Jaime Gómez Rivas[3,*] and Andrea Baldi[1,*]

[1]Department of Physics and Astronomy, Vrije Universiteit Amsterdam, De Boelelaan 1081, 1081 HV Amsterdam, The Netherlands

[2]Department of Physics, Universitas Indonesia, 16424 Depok, Indonesia

[3]Department of Applied Physics and Eindhoven Hendrik Casimir Institute, Eindhoven University of Technology, P.O. Box 513, 5600 MB Eindhoven, The Netherlands

[4]Department of Physics, Chalmers University of Technology, 412 96 Göteborg, Sweden

*Correspondence to: ferryanggoroardynugroho@yahoo.com; clangham@chalmers.se; j.gomez.rivas@tue.nl; a.baldi@vu.nl

Table of Contents

1. Ultrasensitive Hydrogen Sensors State-of-the-Art 3
2. Scanning Electron Microscopy Images of Pd Square Arrays with Different Diameters and Pitch Arrays 4
3. Peak Position and FWHM in Pd Square Array as Function of Pitch Distance and Diameter....5
4. Calculation of Rayleigh Anomalies in Periodic Nanoparticle Array 7
5. Additional Data on the Angle-Dispersion Extinction Spectra of Pd Array 8
6. Sensor Array Parameters Obtained by Particle Swarm Optimization 9
7. Angle Dispersion Extinction and Field Distribution of the Optimized Sensor Array 10
8. Single Particle Counterpart of the Optimized Sensor Array 10
9. Extended Particle Swarm Optimization Method 11
10. Optimized Array Sensor: Structural and Optical Characterization 13
11. Determination of Experimental λ_{peak} and Its Noise....13
12. Other Performance Aspects: Reproducibility, Speed, and Poisoning Resistance 14
13. Optical Properties of Tandem Sensors....16
14. Quasi-Random Array Control Sample: Structural, Optical and Noise Characterization....17
15. Experimental Details on the Gas Flow Used....18

Supplementary References....19

1. Ultrasensitive Hydrogen Sensors State-of-the-Art

In **Table S1** we summarize the available literature on hydrogen sensors with different transduction principles (categorized following the definition given in Ref. [1]) that report sensitivity down to the *ppb* level at room temperature. From the Table, excluding our results here, it is clear that until now, *ppb* sensitivity is only achieved by electrical (and one thermal conductivity) sensors.

Table S1. Ultrasensitive hydrogen sensors operating at room temperature from the literature.

Active Materials[a]	Transducer Platform[b]	LoD[c]	Test Environment[d]	Ref.
Pd@Ni foam	Electrical	0.007	N_2	2
MWCNT	Electrical	0.06	Vacuum	3
Pd NP on Y_2O_3/CNT	Electrical	0.09	N_2	4
Pd NP@CPPy	Electrical	0.1	N_2	5
Pd nanoflower/graphene	Electrical	0.1	N_2	6
PdCo nanohole	Electrical	0.18	Vacuum	7
Pt@PtO_x nanostructures	Electrical	0.2	N.A.	8
Pd NP@PMMA	Optical	0.25	Ar and Air	This work
Pd-Pt NP on ITPES	Electrical	0.4	Air	9
Chalcogenide NW	Thermal conductivity	0.4	Air	10
Ru@CPPy	Electrical	0.5	N_2	11
MoO_3 NR/graphene	Electrical	0.5	Air	12
MoO_3 NR	Electrical	0.5	Air	13
Pd@CNT film	Electrical	0.89	N_2	14
Pd hemispherical NP	Optical	2.5	N_2	15
PdAuCu NP	Optical	7	Vacuum	16
PdAu NP@PTFE/PMMA	Optical	7	Vacuum	17
Pd nanostrip	Optical	10	N_2	18
Pd-Al NR	Optical	40	N_2	19

[a]CNT = carbon nanotubes, CPPy = 3-carboxylated polypyrrole, ITPES = imidazolium-functionalized triptycene polyether sulfone, MWCNT = multi-walled carbon nanotubes, NP = nanoparticles, NR = nanorods, NW = nanowires, PMMA = poly(methyl methacrylate). [b]The category of the transduction mechanism follow the ones defined in Ref. [1]. [c]LoD = limit of detection. Only the works that *explicitly* measured such response at the reported concentration (*i.e.* not extrapolation) are included here. [d]N.A. = Information is not available.

2. Scanning Electron Microscopy Images of Pd Square Arrays with Different Diameters and Pitch Arrays

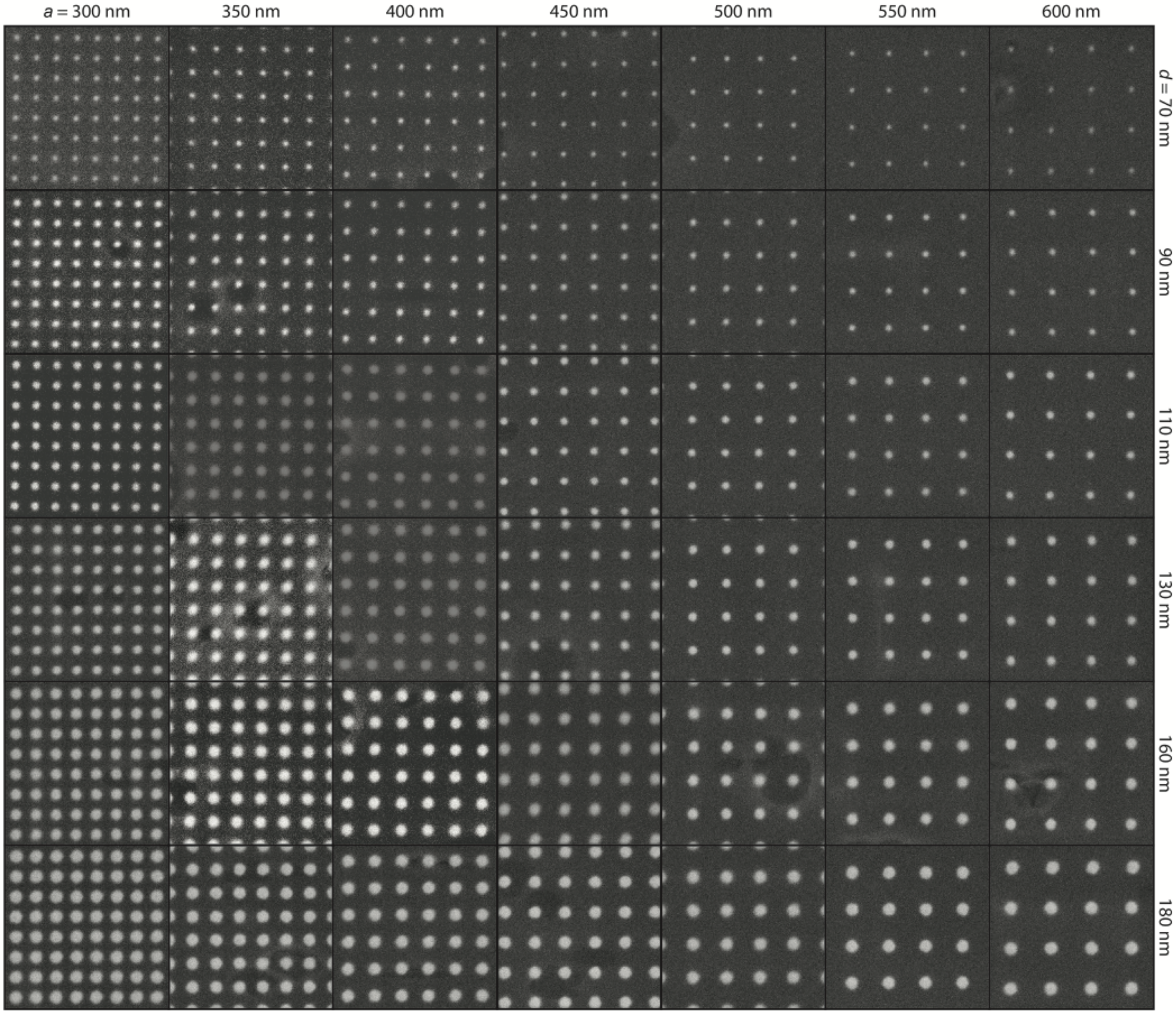

Supplementary Fig. 1. A collage of scanning electron microscopy images of 42 Pd arrays with different diameters and pitch arrays, but with constant height of 45 nm. Note that for imaging purpose, the overlayer PMMA thin film is etched away. Each panel is 24×24 μm^2.

3. Peak Position and FWHM in Pd Square Array as Function of Pitch Distance and Diameter

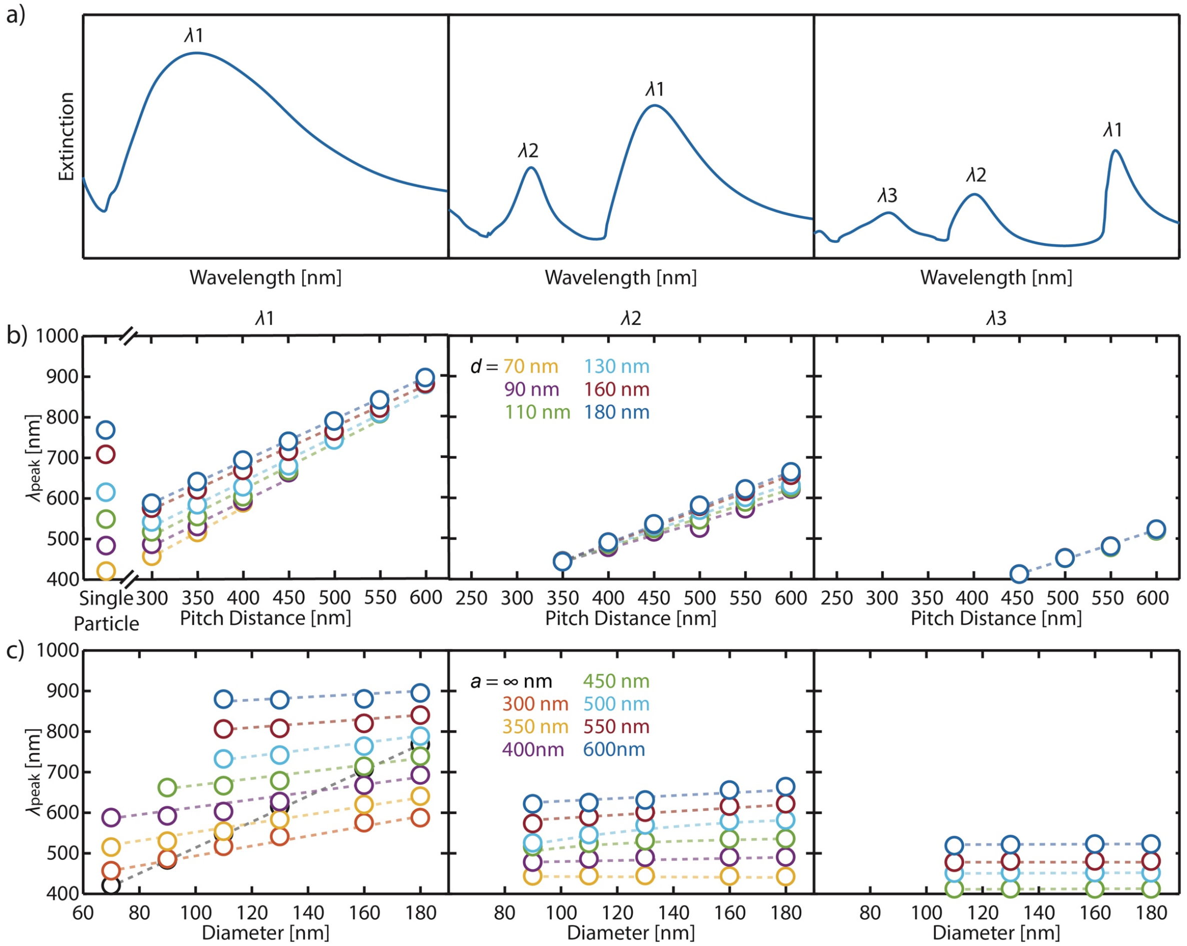

Supplementary Fig. 2. (a) Excerpt of simulated extinction spectra of a Pd square array taken from **Fig. 1** with different numbers of "peaks" (left: $a = 300$ nm, middle: $a = 450$ nm, right: $a = 450$ nm, all with similar $d = 180$ nm, $h = 45$ nm and $t_{PMMA} = 200$ nm). In each case, λ_1 is consistently assigned to the peak at the longest wavelength, followed by λ_2 and λ_3 (the shortest wavelength). (b) Peak position of λ_1 (left), λ_2 (middle) and λ_3 (right) as a function of array pitch distance ($h = 45$ nm, $t_{PMMA} = 200$ nm). Clear dependence of the peak position on the pitch distance in all peaks is observed. (c) Peak position of λ_1 (left), λ_2 (middle) and λ_3 (right) as a function of nanodisk diameter. In contrast to (b), here only λ_1 has dependency on the diameter, whereas λ_2 and λ_3 are rather constant. This behavior corroborates the dominating contribution of the LSPR in λ_1, and of the RA in λ_2 and λ_3, respectively.

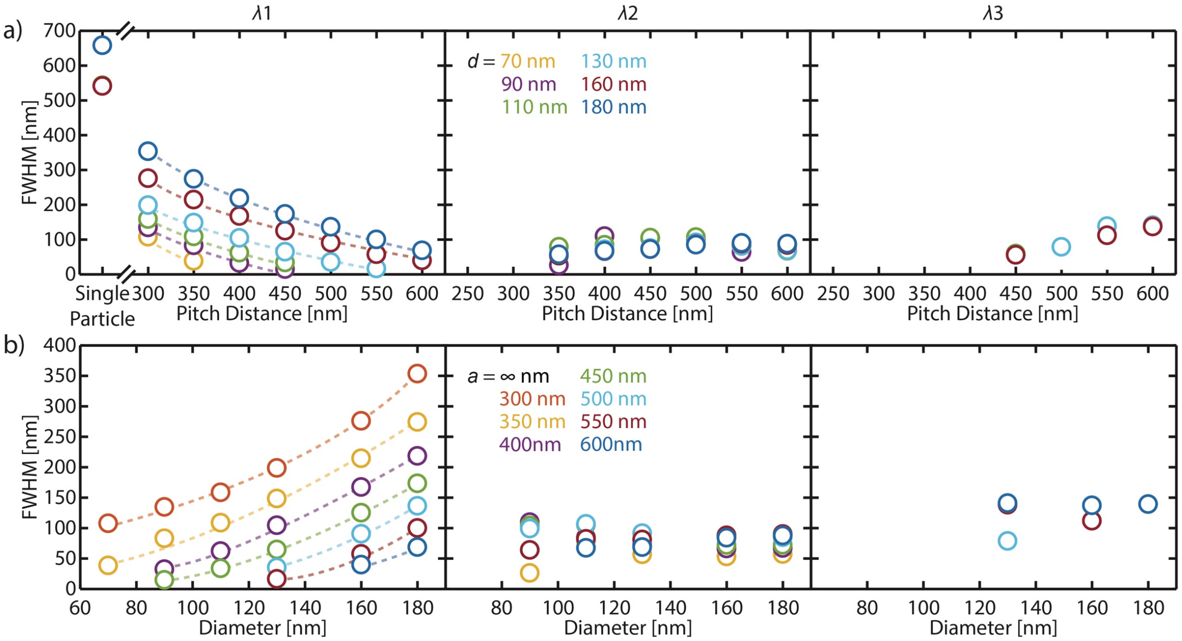

Supplementary Fig. 3. (a) FWHM of λ_1 (left), λ_2 (middle) and λ_3 (right) as a function of array pitch distance (h = 45 nm, t_{PMMA} = 200 nm). Clear dependence of the FWHM on pitch distance in λ_1 is observed, while λ_2 and λ_3 exhibit weak dependency. (b) FWHM of λ_1 (left), λ_2 (middle) and λ_3 (right) as a function of nanodisk diameter. Similar to the case in (a), only λ_1 has dependency on the diameter, whereas λ_2 and λ_3 are rather constant. This behavior, along with the dependency of the peak position shown in **Supplementary Fig. 2**, corroborates the dominating contribution of the LSPR in λ_1, and of the RA in λ_2 and λ_3, respectively.

4. Calculation of Rayleigh Anomalies in Periodic Nanoparticle Array

When light is incident on a 2D square array of subwavelength nanoparticle with pitch distance, *a*, photons can gain additional momentum in integer multiples of

$$|\boldsymbol{G}_x| = |\boldsymbol{G}_y| = 2\pi/a. \tag{S1}$$

Rayleigh anomaly (RA) is associated with light diffracted parallel to the lattice surface, and it occurs when

$$k_{out} = |\boldsymbol{k_0} sin\theta + i\boldsymbol{G}_x + j\boldsymbol{G}_y|. \tag{S2}$$

The wavelength of the RA occurs at the onset of the (i, j) diffraction order, above which free-space light diffraction is forbidden in the order. Here, θ is the incident angle.

In more detail, the different orders of RAs are given by equation:

$$k_{out} = \sqrt{(k_0 sin\theta)^2 + (i^2 + j^2)(2\pi/a)^2 + 2ik_0 sin\theta(2\pi/a)}. \tag{S3}$$

Therefore, when the incident light has polarization of x direction, the wavelengths of (1,0) and (-1,0) orders of RAs are calculated by

$$\lambda_{(\pm1,0)} = \pm a(n - sin\theta), \tag{S4}$$

where n is the refractive index of material at diffracted side and assuming the light is coming from air. Similarly, the wavelength of $(0,\pm1)$ can be expressed as

$$\lambda_{(0,\pm1)} = \sqrt{a^2(n^2 - sin^2\theta)}. \tag{S5}$$

For simplicity, the wavelength or wavevector of higher orders of RAs also can be calculated from Eq. S3.

5. Additional Data on the Angle-Dispersion Extinction Spectra of Pd Array

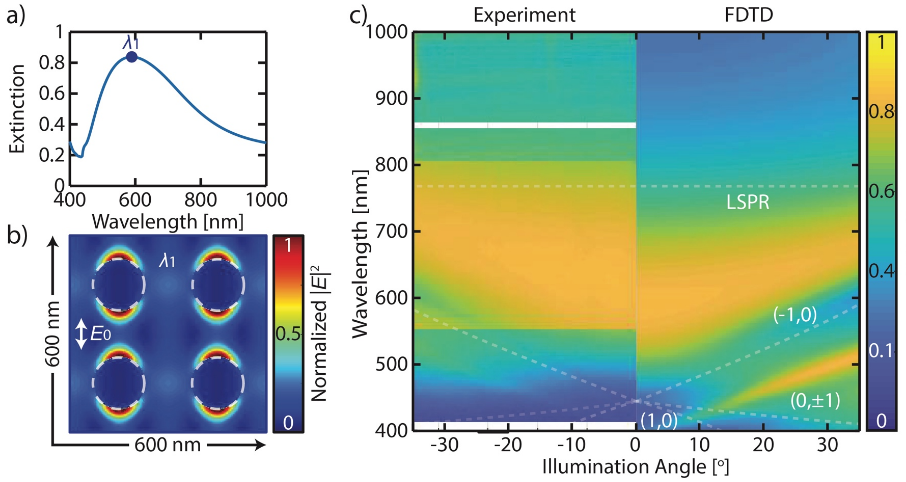

Supplementary Fig. 4. (a) Extinction spectra of a array sample with d = 180 nm, h = 45 nm, a = 300 nm and t_{PMMA} = 200 nm. (b) 2D maps of the normalized total field amplitude $|E|^2$ of the array at wavelength as marked in panel (a) at the mid-height of the nanoparticles. Dashed lines outline the nanodisks. (c) Experimental and simulated wavelength-resolved angle dispersion extinction spectra of the array with d = 180 nm, h = 45 nm, t_{PMMA} = 200 nm and a = 300 nm, showing the different RA orders (dashed lines) and the LSPR position of the corresponding single-particle counterpart.

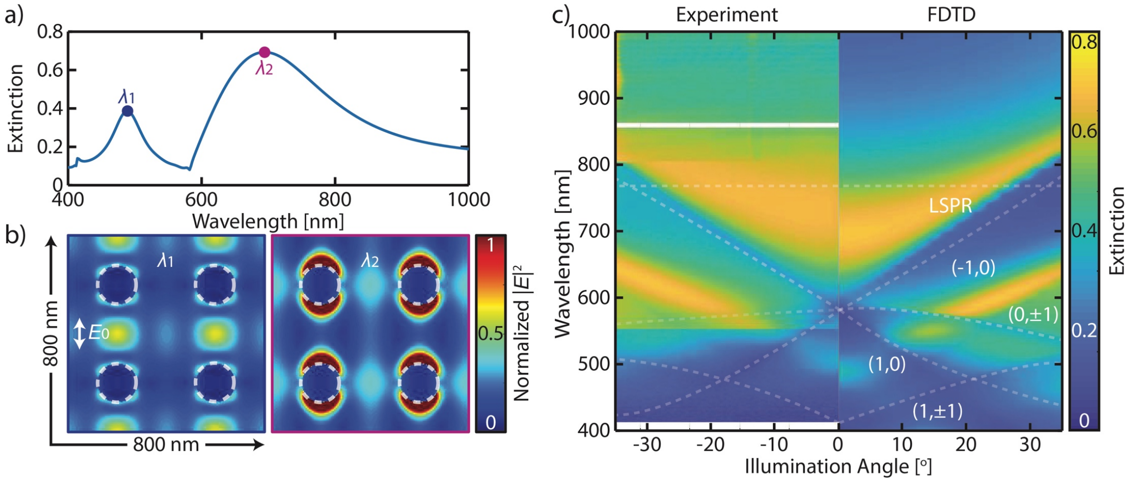

Supplementary Fig. 5. (a) Extinction spectra of a array sample with d = 180 nm, h = 45 nm, a = 400 nm and t_{PMMA} = 200 nm. (b) 2D maps of the normalized total field amplitude $|E|^2$ of the array at different excitation wavelengths, as marked in panel (a) at the mid-height of the nanoparticles. Dashed lines outline the nanodisks. (c) Experimental and simulated wavelength-resolved angle dispersion extinction spectra of the array with d = 180 nm, h = 45 nm, t_{PMMA} = 200 nm and a = 400 nm, showing the different RA orders (dashed lines) and the LSPR position of the corresponding single-particle counterpart.

6. Sensor Array Parameters Obtained by Particle Swarm Optimization

Table S2. Figure-of-Merit and Sensor Array Parameters Obtained by Particle Swarm Optimization after the 15th Generation.

Population	FoM	$\Delta\lambda_{peak}$ [nm]	FWHM [nm]	d [nm]	h [nm]	a [nm]	t_{PMMA} [nm]
1	0.091	63.75	700	300	25	500	161
2	0.079	24.5	310.75	150	20	400	293
3	0.087	19.75	227.25	132	22	438	300
4	0.096	21.75	226.25	120	20	431	300
5	0.082	17.25	211.5	138	20	443	290
6	0.102	28	274.5	121	21	395	297
7	0.095	21.5	227.25	104	23	396	299
8	0.081	20.75	255.5	146	20	429	298
9	0.105	31	296.75	124	20	376	300
10	0.103	30.25	294.5	120	20	376	293

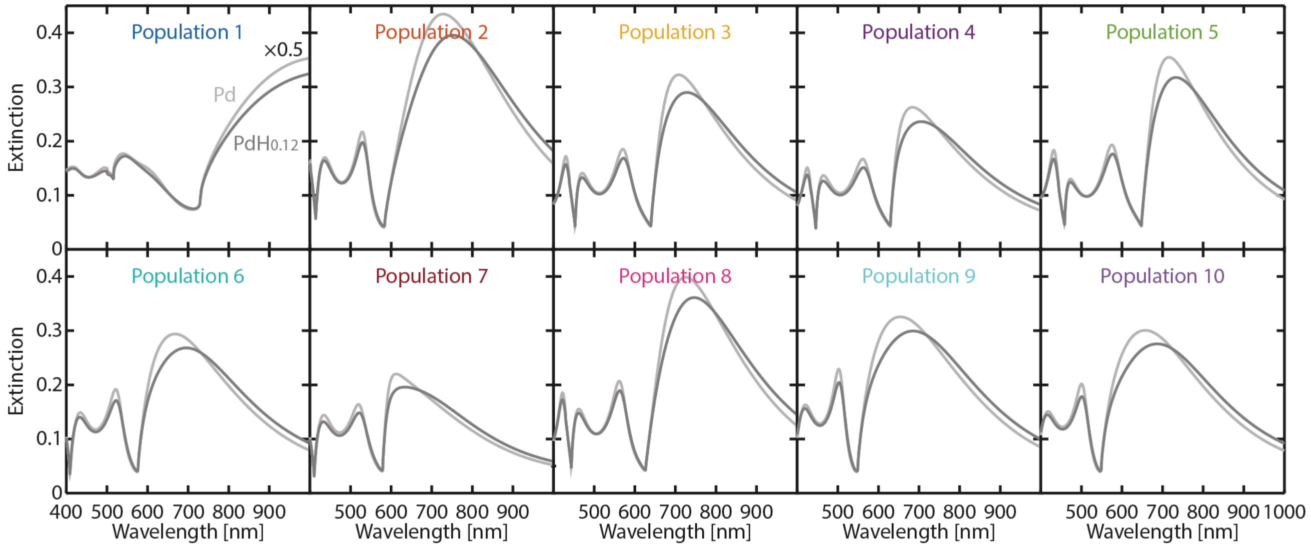

Supplementary Fig. 6. Simulated extinction spectra of the calculated Pd and Pd hydride array obtained by PSO after the 15th generation.

7. Angle Dispersion Extinction and Field Distribution of the Optimized Sensor Array

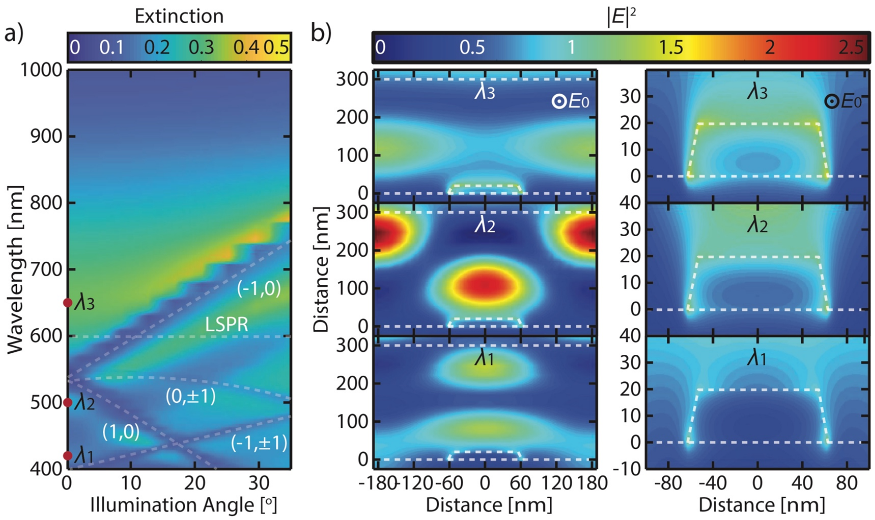

Supplementary Fig. 7. (a) Simulated wavelength-resolved angle dispersion extinction spectra of the optimized sensor array, showing the different RA orders (dashed lines) and the LSPR position of the corresponding single-particle counterpart (**Supplementary Fig. 8**). (b) Field distribution surrounding the nanoparticle (left) and at the close vicinity and inside of the nanoparticle (right) at three different excitation wavelengths corresponding to the extinction peaks (λ_1-λ_3). From the maps it is clear that relative field amplitude *inside* the nanoparticle excited at λ_1 and λ_2 are lower than the one at λ_3. This again corroborates the nature of the peak, in which λ_3 is dominated by the LSPR and thus is sensitive to the change from Pd to Pd hydride. Dashed lines outline the interfaces between glass/nanodisks/PMMA/air.

8. Single Particle Counterpart of the Optimized Sensor Array

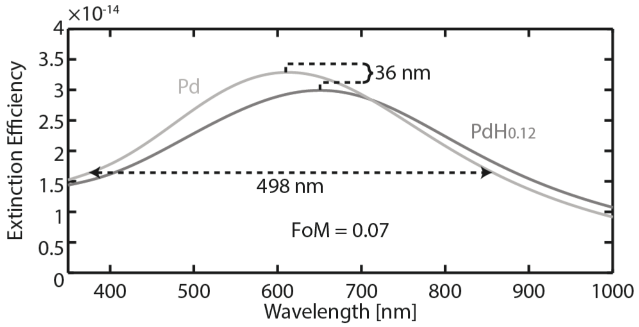

Supplementary Fig. 8. Calculated Pd and $PdH_{0.12}$ extinction spectra of a single Pd nanodisk with same geometrical parameters to the optimized array sensor obtained by PSO (d = 124 nm, h = 20 nm, a = 376 nm, t_{PMMA} = 300 nm). Comparable peak shift as for the array sensor is found upon hydrogenation but with very broad FWHM. This combination results in a FoM of 0.07.

9. Extended Particle Swarm Optimization Method

If we assume the position of the $Pd/PdH_{0.12}$ in the parameter space as a function of four variables, $x_i(n) = f(d(d), h(n), a(n), t_{PMMA}(n))$, where *d(n), h(n), a(n) and* $t_{PMMA}(n)$ are the particle diameter, height and the pitch distance of the array, t_{PMMA} is the thickness of PMMA, and n is the generation counter. During the optimizing process, the particle is subjected to three forces as it moves through the parameter spaces: (i) a frictional force that is proportional to the velocity, $\alpha v_i(n)$, where α is the inertial weight; (ii) a spring force towards the individual best value of this particle, $c_1 r_1[\hat{x}_i(n) - x_i(n)]$, where c_1 is the cognitive factor and r_1 is a random number between 0 and 1; and (iii) a spring force towards the global best value of all the particles, $c_2 r_2[g(n) - x_i(n)]$, where c_2 is the social factor and r_2 is a random number between 0 and 1.

The velocity in the next generation can be obtained from the sum of these three forces, *i.e.*, $v_i(n + 1) = \alpha v_i(n) + c_1 r_1[\hat{x}_i(n) - x_i(n)] + c_2 r_2[g(n) - x_i(n)]$. In the Lumerical solver, we used the default values of $c_1 = c_2 = 1.49$ and linearly spaced values of α between minimum 0.4 and maximum 0.9 for PSO simulations that have been verified to converge well in many test optimization for photonic design problems. The position of the particle in the next generation is then given by $x_i(n + 1) = x_i(n) + v_i(n + 1)$.

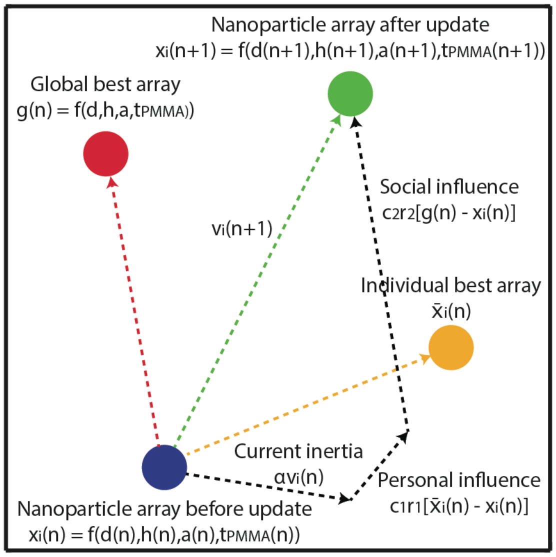

Supplementary Fig. 9. PSO algorithm for one nanoparticle array in the PSO terminology updating during one generation.

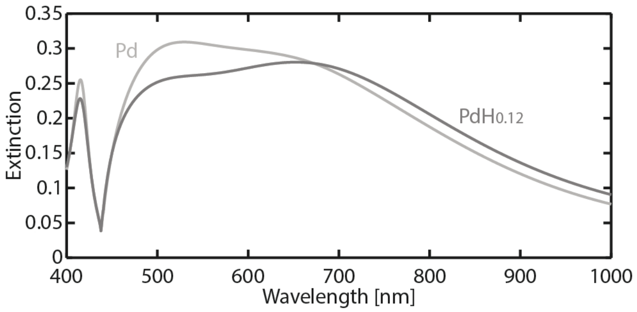

Supplementary Fig. 10. Simulated extinction spectra of Pd and $PdH_{0.12}$ for Population 2 after 18th generation of optimization. Clearly the PSO reaches a configuration where the two SLR peaks are close to each other and thus indistinguishable. Due to this condition, when the spectra change from Pd to Pd hydride, the program assigns a different peak, which then results in seemingly large peak shift (and thus falsely large FoM). To avoid this problem, we stopped our PSO simulation at the 15th generation.

10. Optimized Array Sensor: Structural and Optical Characterization

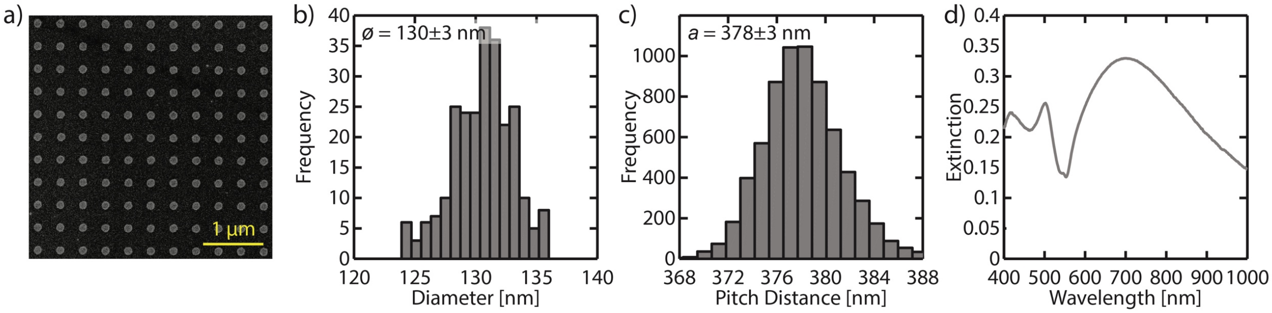

Supplementary Fig. 11. (a) SEM image of the sensor array. (b) Diameter distribution of the particles forming the sensor, showing an average of 130 nm, which is slightly larger than the targeted diameter of 124 nm. (c) Pitch distance distribution of the sensor array with average of 378 nm, which is very close to the targeted 376 nm. (d) Experimental extinction spectra of the fabricated array sensor.

11. Determination of Experimental λ_{peak} and Its Noise

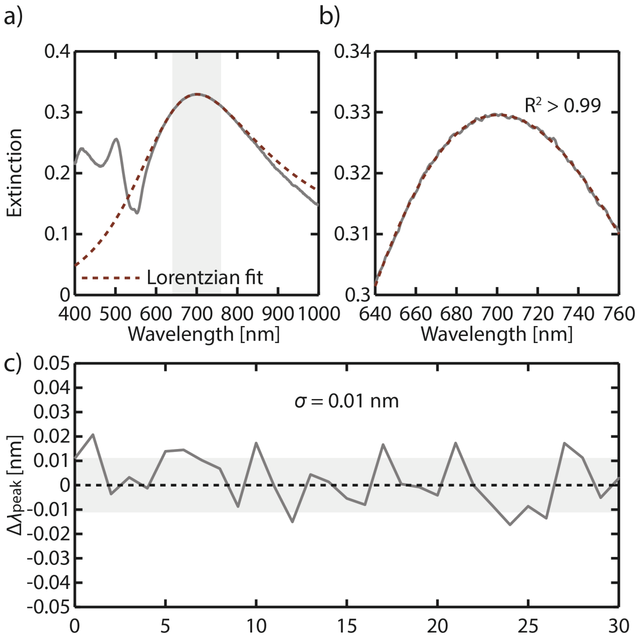

Supplementary Fig. 12. (a) Lorentzian function fitting (red dashed line) to the experimental optical spectra to extract λ_{peak}. In our analysis, the fit is only applied within ±60 nm from the peak maximum (grey shaded area, following the method established in Ref. [17]), where the peak is symmetric. (b) Zoomed-in version of (a) within the fit range. Clearly, the Lorentzian represents well the data and thus enables a good fit with $R^2 > 0.99$. (c) Lorentzian-fitted $\Delta\lambda_{peak}$ response of the best sensor (*cf.* **Fig. 4**a) in the first 30 min of operation used to derive the peak-to-peak readout noise, σ, of 0.01 nm. The dashed lines and gray-shaded areas denote the mean of the signal and $\pm\sigma$ from the mean, respectively.

12. Other Performance Aspects: Reproducibility, Speed, and Poisoning Resistance

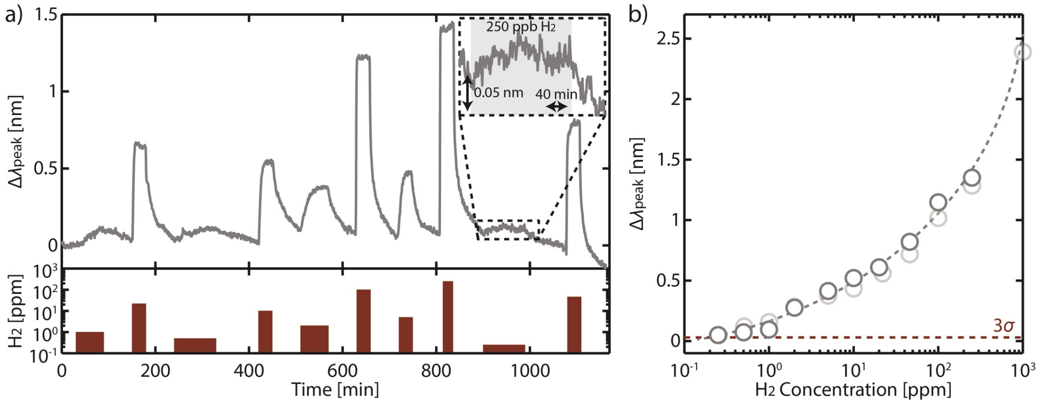

Supplementary Fig. 13. (a) $\Delta\lambda_{peak}$ response to stepwise random H_2 concentration (250 to 0.25 *ppm*) in Ar carrier gas at room temperature. Inset: zoomed-in version of the sensor response to 250 *ppb* H_2. (b) Measured $\Delta\lambda_{peak}$ as a function of H_2 concentration derived from (a). The transparent symbols and gray dashed line are reproduced from **Fig. 4**b. The sensor's responses to these random H_2 exposure are consistent with the descending one, and thus exemplifying the reproducibility of the sensor.

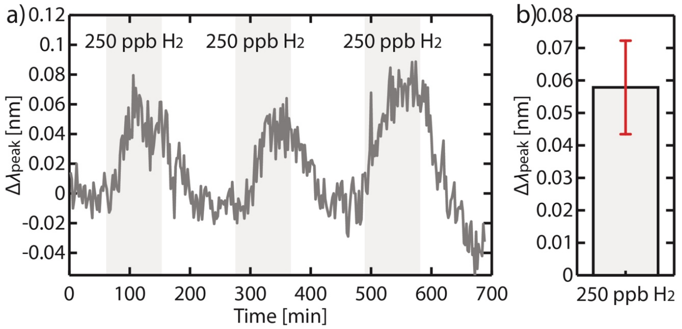

Supplementary Fig. 14. (a) $\Delta\lambda_{peak}$ response to three consecutive cycles of 250 *ppb* H_2 (grey areas). A reversible and reproducible sensor response to such low concentration of H_2 is observed. (b) Average sensor signal to the three cycles of 250 *ppb* H_2 exposure. An uncertainty of ~0.01 nm is recorded, which is in the same order of the sensor's signal noise.

To deduce the response and recovery times of the sensors we use the commonly used t_{90} and t_{10}, respectively (**Supplementary Fig. 15**a-b). As plotted in **Supplementary Fig. 15**c-d, the response and recovery times of the sensor increase with the lowering H_2 concentration. Such observation is inherent to Pd nanostructures as previously shown.[15,17,20] As a result, at the lowest H_2 concentration of 250 *ppb*, the sensor's response time is in the order of 40 min. Interestingly, the response and recovery times of the control random array sensor are practically similar to the optimized one (**Supplementary Fig. 15**c-d). Such finding reveals that our method of increasing the sensor's sensitivity *via* periodic arrangement *does not* affect its sensing speed as it is mainly defined by the materials design. Thus, our sensitivity improvement method can be combined with other methods aimed to directly enhance the sensor's speed, for example by employing nanoparticles with reported faster kinetics than Pd (*e.g.* PdAu,[17,21] PdCo[15] and PdTa[22] alloys, with speed twice as fast compared to pure Pd) or by utilizing polymer coatings with higher kinetics-enhancements such as PTFE (twice as high as PMMA).[17]

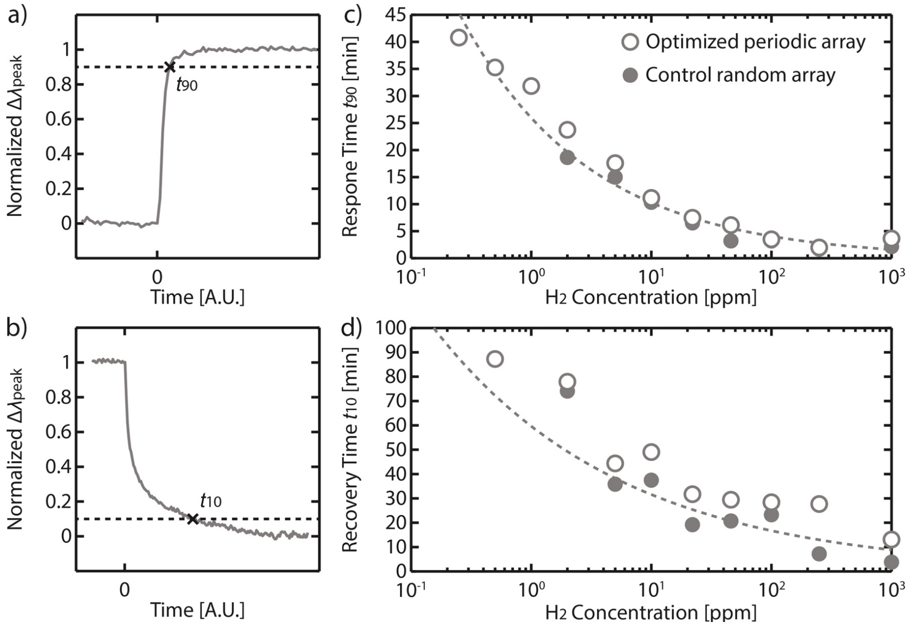

Supplementary Fig. 15. The definition of (a) response time as t_{90} and (b) recovery times as t_{10}, which correspond to the time it takes to reach 90% and 10% of the normalized signal (with respect to signal during the exposure and in the absence of H_2), respectively. (c) Response times and (d) recovery times of the optimized periodic array sensor and control random array sensor as function of H_2 concentration. Data is extracted from **Fig. 4**a and c, respectively. The recovery and response times of both sensors are comparable and can practically be described with a single trend (the dashed lines), as established in ref. [17].

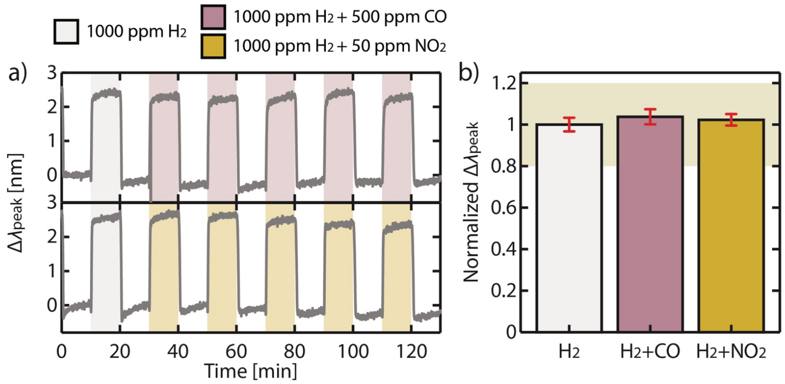

Supplementary Fig. 16. (a) Time-resolved $\Delta\lambda_{peak}$ response of sensor 1 pulse of 1000 *ppm* H_2 followed by 5 pulses of 1000 *ppm* H_2 + 500 *ppm* CO, and 1000 *ppm* H_2 + 50 *ppm* NO_2 in Ar. (b) Normalized sensor signal to the one obtained in 1000 *ppm* H_2. The error bars denote the standard deviation from 5 cycles. The shaded area indicates the ±20% deviation limit from the normalized $\Delta\lambda_{peak}$ in 1000 *ppm* H_2.

13. Optical Properties of Tandem Sensors

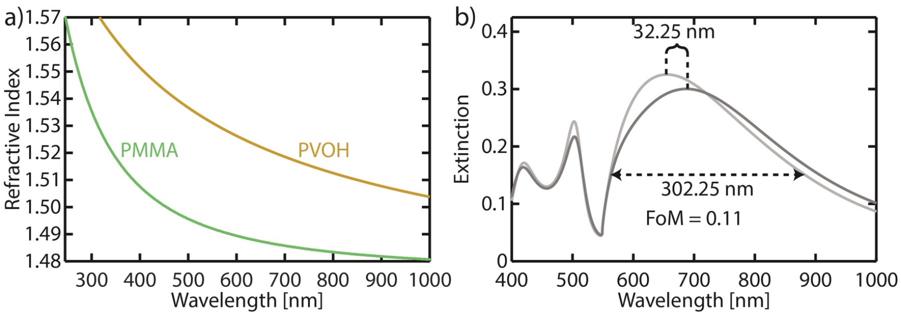

Supplementary Fig. 17. (a) Experimental refractive indices of PMMA and PVOH. The data of PMMA is reproduced from [23]. (b) Unit cell schematic the tandem sensor (d = 124 nm, h = 20 nm, a = 376 nm), with t_{PMMA} = 295 nm and t_{PVOH} = 5 nm. (b) FDTD-calculated extinction spectra of tandem sensor (see the schematic in **Fig. 5**b) for Pd (light gray) and $PdH_{0.12}$ (dark grey) nanodisk arrays. The spectra are basically identical to the ones of the sensor coated with 300 nm PMMA (*cf.* **Fig. 3**d).

14. Quasi-Random Array Control Sample: Structural, Optical and Noise Characterization

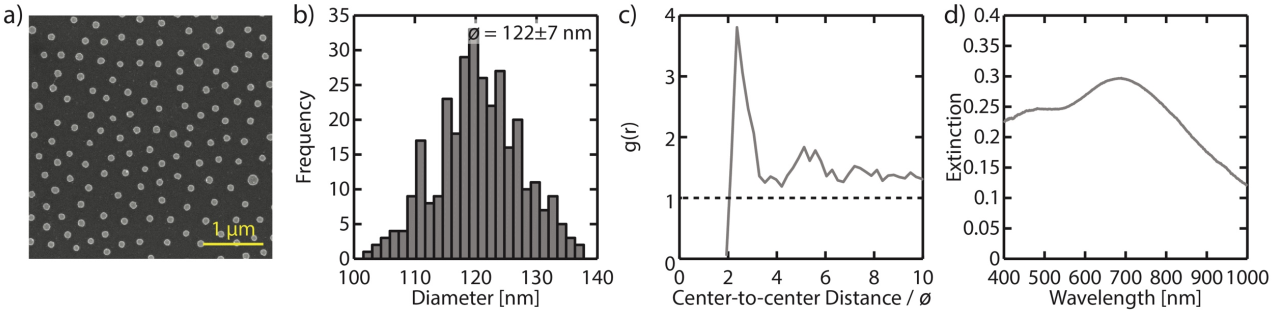

Supplementary Fig. 18. (a) SEM image of the control sensor array with quasi-random particle distribution. (b) Diameter distribution of the particles forming the sensor, showing an average of 122 nm, which is slightly smaller than the targeted diameter of 124 nm. (c) Radial distribution function (RDF) of the control sensor. The primary peak in the RDF (*i.e.*, ~2.5⌀) indicates the average center-to-center distance between neighboring nanostructures. (d) Experimental extinction spectra of the quasi-random array.

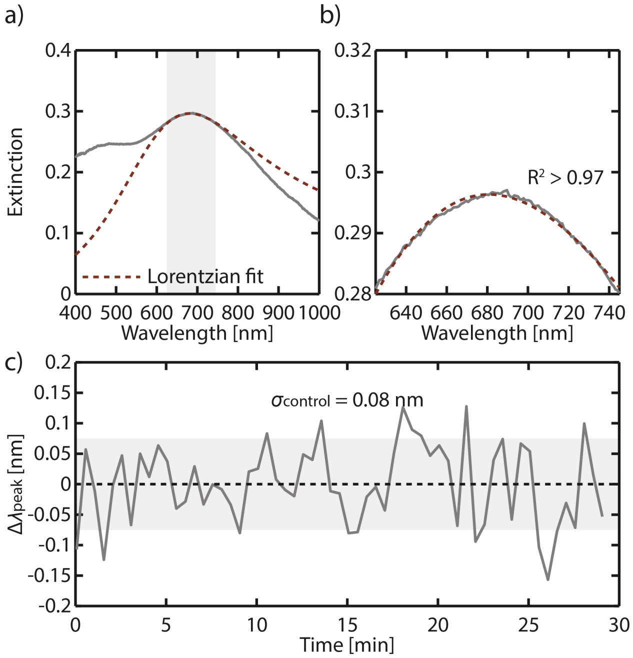

Supplementary Fig. 19. (a) Lorentzian function fitting (red dashed line) within ±60 nm from the peak maximum (grey shaded area) to extract λ_{peak}. (b) Zoomed-in version of (a) within the fit range. The Lorentzian represents the data well in the peak-maximum region and thus enables a good fit with $R^2 > 0.97$. (c) Lorentzian-fitted $\Delta\lambda_{peak}$ response of the quasi-random array control sensor (*cf.* **Fig. 4**c). The derived peak-to-peak noise, $\sigma_{control}$, is 0.08 nm, much higher than that of the optimized regular array sensor. The dashed lines and gray-shaded areas denote the mean of the signal and $\pm\sigma$ from the mean, respectively.

15. Experimental Details on the Gas Flow Used

Table S3. Set Flow of Hydrogen and Argon Gas to Achieve the Targeted Hydrogen Concentrations.

Targeted H_2 Concentration [*ppm*]	1000 *ppm* H_2 Flow [mL/min]	Ar Flow [mL/min]
1000	1	0
250	5	15
100	2	18
46	1	21
22	1	44
10	1	99
5	0.5	199
2	0.25	125
1	0.1	100
0.75	0.1	133
0.5	0.1	200
0.25	0.05	200

Supplementary References

1. Hübert, T., Boon-Brett, L., Black, G. & Banach, U. Hydrogen sensors - A review. *Sensors and Actuators B: Chemical* vol. 157 329–352 (2011).

2. Tian, J. *et al.* A Ppb-level hydrogen sensor based on activated Pd nanoparticles loaded on oxidized nickel foam. *Sensors and Actuators, B: Chemical* **329**, 129194 (2021).

3. Darabpour, M. & Doroodmand, M. M. Fabrication of a glow discharge plasma-based ionization gas sensor using multiwalled carbon nanotubes for specific detection of hydrogen at parts per billion levels. *IEEE Sensors Journal* **15**, 2391–2398 (2015).

4. Zhou, S. *et al.* Sub-10 parts per billion detection of hydrogen with floating gate transistors built on semiconducting carbon nanotube film. *Carbon N Y* **180**, 41–47 (2021).

5. Lee, J. S., Kim, S. G., Cho, S. & Jang, J. Porous palladium coated conducting polymer nanoparticles for ultrasensitive hydrogen sensors. *Nanoscale* **7**, 20665–20673 (2015).

6. Shin, D. H. *et al.* Flower-like Palladium Nanoclusters Decorated Graphene Electrodes for Ultrasensitive and Flexible Hydrogen Gas Sensing. *Scientific Reports* **5**, 12294 (2015).

7. Pham, M. T. *et al.* Pd80Co20 Nanohole Arrays Coated with Poly(methyl methacrylate) for High-Speed Hydrogen Sensing with a Part-per-Billion Detection Limit. *ACS Applied Nano Materials* **13**, (2021).

8. Basu, P. K., Kallatt, S., Anumol, E. A. & Bhat, N. Suspended core-shell Pt-PtOx nanostructure for ultrasensitive hydrogen gas sensor. *Journal of Applied Physics* **117**, 224501 (2015).

9. Koo, W.-T., Kim, Y., Kim, S. & Lee, S.-J. Hydrogen Sensors from Composites of Ultra-small Bimetallic Nanoparticles and Porous Ion- Exchange Polymers. *Chem* (2020) doi:10.1016/j.chempr.2020.07.015.

10. Kim, S. *et al.* Thermochemical hydrogen sensor based on chalcogenide nanowire arrays. *Nanotechnology* **26**, 145503 (2015).

11. Oh, J., Lee, J. S. & Jang, J. Ruthenium decorated polypyrrole nanoparticles for highly sensitive hydrogen gas sensors using component ratio and protonation control. *Polymers (Basel)* **12**, 1427 (2020).

12. Yang, S. *et al.* Remarkably accelerated room-temperature hydrogen sensing of MoO3 nanoribbon/graphene composites by suppressing the nanojunction effects. *Sensors and Actuators B: Chemical* **248**, 160–168 (2017).

13. Yang, S. *et al.* Highly Responsive Room-Temperature Hydrogen Sensing of α-MoO3 Nanoribbon Membranes. (2015) doi:10.1021/ACSAMI.5B01858.

14. Xiao, M. *et al.* Batch Fabrication of Ultrasensitive Carbon Nanotube Hydrogen Sensors with Sub-ppm Detection Limit. *ACS Sensors* **3**, 749–756 (2018).

15. Luong, H. M. *et al*. Sub-second and ppm-level optical sensing of hydrogen using templated control of nano-hydride geometry and composition. *Nature Communications* **12**, 2414 (2021).

16. Darmadi, I., Nugroho, F. A. A., Kadkhodazadeh, S., Wagner, J. B. & Langhammer, C. Rationally Designed PdAuCu Ternary Alloy Nanoparticles for Intrinsically Deactivation-Resistant Ultrafast Plasmonic Hydrogen Sensing. *ACS Sensors* **4**, 1424–1432 (2019).

17. Nugroho, F. A. A. *et al*. Metal–polymer hybrid nanomaterials for plasmonic ultrafast hydrogen detection. *Nature Materials* **18**, 489–495 (2019).

18. He, J. *et al*. Integrating plasmonic nanostructures with natural photonic architectures in Pd-modified: Morpho butterfly wings for sensitive hydrogen gas sensing. *RSC Advances* **8**, 32395–32400 (2018).

19. Yue, S. *et al*. CMOS-compatible plasmonic hydrogen sensors with a detection limit of 40 ppm. *Optics Express* **27**, 19331 (2019).

20. Koo, W. T. *et al*. Accelerating Palladium Nanowire H2 Sensors Using Engineered Nanofiltration. *ACS Nano* **11**, 9276–9285 (2017).

21. Wadell, C. *et al*. Hysteresis-Free Nanoplasmonic Pd-Au Alloy Hydrogen Sensors. *Nano Letters* **15**, 3563–3570 (2015).

22. Bannenberg, L., Schreuders, H. & Dam, B. Tantalum-Palladium: Hysteresis-Free Optical Hydrogen Sensor Over 7 Orders of Magnitude in Pressure with Sub-Second Response. *Advanced Functional Materials* 2010483 (2021) doi:10.1002/adfm.202010483.

23. Nugroho, F. A. A., Albinsson, D., Antosiewicz, T. J. & Langhammer, C. Plasmonic Metasurface for Spatially Resolved Optical Sensing in Three Dimensions. *ACS Nano* **14**, 2345–2353 (2020).
